# Quasi-periodic spatiotemporal models of brain activation in single-trial MEG experiments

**DOI:** 10.1177/1471082X14524673

**Published:** 2014-10

**Authors:** Massimo Ventrucci, Adrian W Bowman, Claire Miller, Joachim Gross

**Affiliations:** 1 Department of Statistical Sciences, University of Bologna, Bologna, Italy; 2 School of Mathematics and Statistics, University of Glasgow, Glasgow, UK; 3 Centre for Cognitive Neuroimaging, University of Glasgow, Glasgow, UK

**Keywords:** quasi-periodic, spatiotemporal, p-splines, MEG, magneto-encephalography

## Abstract

Magneto-encephalography (MEG) is an imaging technique which measures neuronal activity in the brain. Even when a subject is in a resting state, MEG data show characteristic spatial and temporal patterns, resulting from electrical current at specific locations in the brain. The key pattern of interest is a ‘dipole’, consisting of two adjacent regions of high and low activation which oscillate over time in an out-of-phase manner. Standard approaches are based on averages over large numbers of trials in order to reduce noise. In contrast, this article addresses the issue of dipole modelling for single trial data, as this is of interest in application areas. There is also clear evidence that the frequency of this oscillation in single trials generally changes over time and so exhibits quasi-periodic rather than periodic behaviour. A framework for the modelling of dipoles is proposed through estimation of a spatiotemporal smooth function constructed as a parametric function of space and a smooth function of time. Quasi-periodic behaviour is expressed in phase functions which are allowed to evolve smoothly over time. The model is fitted in two stages. First, the spatial location of the dipole is identified and the smooth signals characterizing the amplitude functions for each separate pole are estimated. Second, the phase and frequency of the amplitude signals are estimated as smooth functions. The model is applied to data from a real MEG experiment focusing on motor and visual brain processes. In contrast to existing standard approaches, the model allows the variability across trials and subjects to be identified. The nature of this variability is informative about the resting state of the brain.

## 1 Introduction

**Figure 1:**
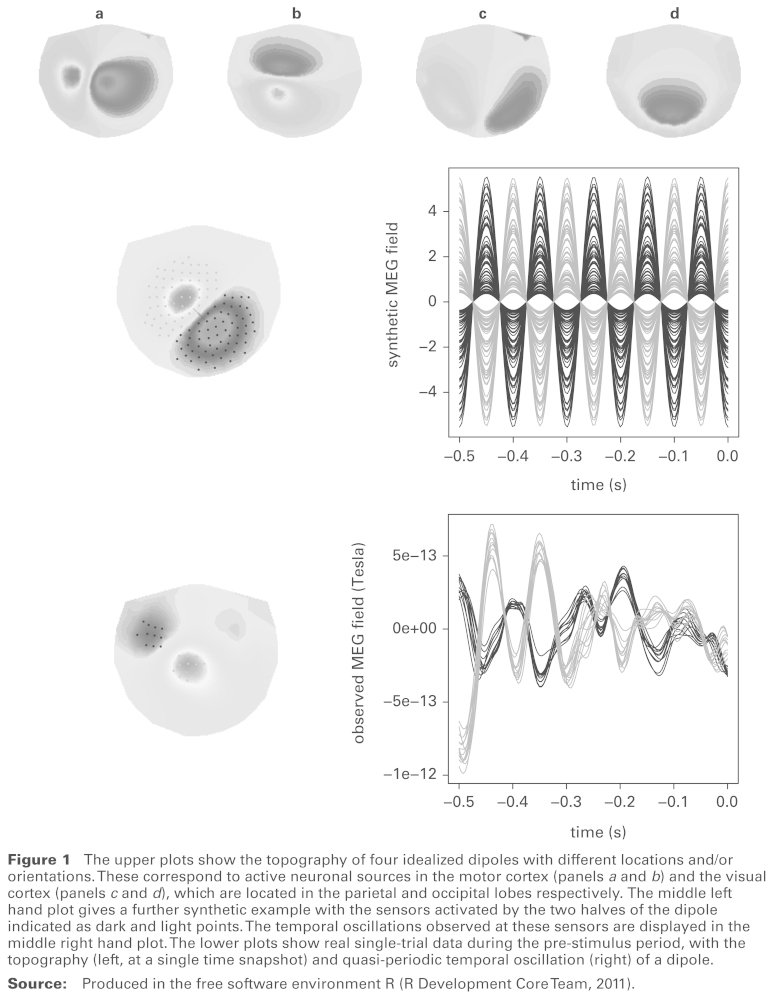
The upper plots show the topography of four idealized dipoles with different locations and/or orientations. These correspond to active neuronal sources in the motor cortex (panels *a* and *b*) and the visual cortex (panels *c* and *d*), which are located in the parietal and occipital lobes respectively. The middle left hand plot gives a further synthetic example with the sensors activated by the two halves of the dipole indicated as dark and light points. The temporal oscillations observed at these sensors are displayed in the middle right hand plot. The lower plots show real single-trial data during the pre-stimulus period, with the topography (left, at a single time snapshot) and quasi-periodic temporal oscillation (right) of a dipole. **Source:** Produced in the free software environment R (R Development Core Team, 2011).

Magneto-encephalography (MEG) data record measurements of the magnetic field outside the head with the aim of detecting the electrical activation of neurons in the brain. Hämäläinen *et al.* (1993) give a description of MEG imaging. Data are collected by around 200–300 sensitive sensor devices embedded in a helmet placed over the head of the patient. These observations are usually available at a high temporal resolution of around 4ms and moderate spatial resolution, with sensor spacing around 2-3cm. However, the magnetic and electrical nature of the process can produce substantial noise in the data. A general aim in MEG experiments is to study brain activation associated with particular experimental conditions where participants are exposed to sensory stimuli. In many cases, the resulting activation can be modelled as point-like current sources that produce a characteristic magnetic field pattern outside the head, consisting of two adjacent spatial regions with oppositely signed fields; see, for example, Hämäläinen *et al.* (1993). Four idealized examples of this are given in the top row of Figure 1, using information provided in the FieldTrip software described by Oostenveld *et al.* (2011). Here the surface of the helmet has been flattened into a two-dimensional representation, with the face at the top. In addition to this characteristic spatial pattern, activated brain areas often show a characteristic temporal pattern consisting of periodic oscillations. The underlying spatiotemporal MEG signal associated with this type of brain activation is referred to as a ‘dipole’. The middle left panel of Figure 1 gives an example of the spatial pattern (or topography) of a dipole, with a line superimposed to show the distance between the two oppositely-signed ‘poles’. The two-dimensional location of the dipole pattern is identified by the mid-point of this line and its orientation by the perpendicular angle (corresponding to current flow). The middle right hand panel of Figure 1 shows idealized temporal behaviour of the dipole at the highlighted sensors, with periodic oscillation.

MEG data can exhibit substantial noise and this is commonly addressed by taking measurements from a large number of repeated trials from each subject. It is then standard practice to employ some form of averaging across trials in order to reduce the noise in the data. For example, signal processing methods can be used to identify the dominant frequency at a particular sensor, averaged over trials, and the resulting spatial pattern of frequency inspected. The three-dimensional location of a dipole within the brain can then be estimated from the MEG data observed on the surface of the scalp by the solution of an inverse problem, as discussed by Mosher *et al.* (1992), Darvas *et al.* (2004) and many later authors. A recent example of work directed at dipole models and the associated inverse problem is Tian *et al.* (2012).

However, the isolation of particular features such as frequency may lose valuable information in its simplification of a complex spatiotemporal pattern, and the use of averaging also risks the danger of blurring the within-trial variation of spatial features. In contrast, there is particular interest in the alertness status which the brain retains when it is attending to visual stimuli, as described by Van Dijk *et al.* (2008) and Thut *et al.* (2006) and a number of studies, such as those described by Liu and Ioannides (1996) and Mustaffa *et al.* (2010), have provided evidence that variations in the state of the brain at stimulus presentation may influence some of the signal components of the response. In other words, some of the variation across trials in the MEG response to a stimulus may be explained by the variation in brain-activation during the pre-stimulus period. Models of pre-stimulus brain activation may therefore provide useful information for the modelling of post-stimulus activation. The general issue of trial-to-trial variability has been the focus of several papers which address methods for the analysis of single-trial data; see, for example, Quiroga and Garcia (2003) and Ventrucci *et al.* (2011). The models developed below therefore focus on the analysis of, and variation in, single trial data during the pre-stimulus period.

A focus on single trial data introduces several issues. The first is the substantial noise inherent in single replicates. Smoothing techniques can ameliorate this, as discussed by Ventrucci *et al.* (2011). A second issue is that inspection of single trial data suggests that, while the spatial location of a dipole is likely to be reasonably constant for each trial, there is clear evidence of changes in dipole frequency over time. This may be indicative of changes in brain activity which are of interest and a model which can accommodate changes of this type is therefore necessary. This is illustrated on real data (described in Section 2) in the lower panels of Figure 1. The spatial characteristics of a dipole are evident in the lower left panel and its oscillations tracked in the lower right panel. From the latter plot, it is clear that the description of dipole behaviour over time needs to allow quasi-periodic rather than strictly periodic patterns. A third issue is that modelling at the single trial level then allows variability across trials to be investigated. Insights of this type are not available from standard methods of averaging based on an assumption of stationarity.

These considerations require a model for the occurrence of dipoles over the pre-stimulus period at the single trial level. The MEG literature is able to provide useful guidance on the pre-stimulus period, with dipoles expected in the visual and motor cortex with oscillations in the range 8–12 Hz, referred to as the α-band frequency; see Schnitzler and Gross (2005). A quasi-periodic spatiotemporal smooth dipole model is introduced in Section 3. The main aim of the model is to characterize dipoles through a small number of flexible and informative parameters. This includes simple quantities such as location and orientation as well as functional parameters which describe smooth changes in frequency (or phase) and amplitude over time. A two-stage fitting algorithm for this model is described in Section 4 and its performance is evaluated in a simulation study reported in Section 5. The results of applying the model to real MEG data are presented in Section 6, where very interesting insights into trial variability are provided. All of this work is conducted in the two-dimensional space of the scalp surface, avoiding the need to solve the inverse problem to identify dipole location in the three-dimensional brain. The article closes with further discussion in Section 7.

## 2 Experimental data

The purpose of the experiment discussed here was to study information transfer from visual to motor areas and to investigate to what extent reaction time is determined by the brain state prior to stimulus presentation. A stimulus in the form of a light appearing on a screen was used to prompt activation in the visual cortex of the brain.

Nineteen participants were asked to focus on a fixation cross presented in the centre of the screen. An arrowhead pointing to the left or right was added to the fixation cross and participants could be asked to respond with either a left or right index finger button-press, giving a combination of four experimental conditions. Perception of the light stimulus is associated with activation of the visual cortex while finger movement is associated with activation of the motor cortex. As is common in this type of experimental protocol, around one hundred replicates (or trials) were undertaken for each experimental condition. The order of presentation of the experimental conditions was randomized across the full sequence of trials in order to avoid bias due to learning effects.

In a single trial of this experiment, the MEG field was measured at 248 sensors, with one observation every 4ms, for a time lasting one second in total. The first half second, from –500ms to 0ms, corresponds to a pre-stimulus period which is the main focus of this article. A stimulus onset at 0ms is followed by a post-stimulus monitoring period of half a second, from 0 to 500ms.

## 3 Spatiotemporal smooth dipole model

When a neuronal source located inside the brain is active we expect MEG signals to exhibit the structure of a smooth function of both space and time, following a dipole pattern, corrupted by random noise. The spatial smooth component of the dipole consists of two ellipsoidal regions located close to each other. Figure 1 shows four idealized examples of MEG topographies. The dipole is located in the motor cortex in panels *a* and *b* and in the visual cortex in panels *c* and *d*. Within each pair, the dipole has the same location but different orientations. In panel *d*, the orientation of the dipole current means that only one pole of the magnetic field lies within the region captured by the MEG sensors. The model described below can in principle be adapted to accommodate this situation but the focus will be on the much more common situation where both parts of the dipole lie within the sensor region.

When a dipole is present, the mean brain map *m*(*x*, *y*, *t*) over spatial locations *x*, *y* and time *t* can be described as the sum of two spatiotemporal smooth functions, one for each pole. Under the reasonable assumption that the location and orientation of the dipole is fixed for a particular trial, this can be structured as:

(3.1) $$m(x,y,t){}_{1}(x,y){}_{1}(t){}_{2}(x,y){}_{2}(t)$$

where the smooth functions ${}_{1}(x,y)$, ${}_{2}(x,y)$ define the topography of the two poles and the smooth functions ${}_{1}(t)$, ${}_{2}(t)$ describe the oscillating pattern of the poles over time. It is reasonable to assume that outside the region of influence of the dipole the mean signal is 0 and so no intercept term is required in the model.

### 3.1 Modelling the spatial pattern

Figure 1 shows that idealized dipoles exhibit relatively simple, smooth shapes. Interest lies in the key characteristics of location, orientation and size and not in the very detailed features of the dipole topography. From this perspective, and supported by inspection of observed dipole patterns, a very simple but effective model for dipole topography is proposed by giving each pole the shape of a (scaled) bivariate normal density function:

(3.2) $${}_{i}(x,y)\exp\left\{\frac{1}{2h^{2}}\mathrm{\{(}x{}_{x,i}{)}^{2}(y{}_{y,i}{)}^{2}\}\right\}\;\;\;i1,2,$$

with location determined by $({}_{x,i},{}_{y,i}{)}^{}$ and radial size by *h*, where *i* = 1, 2 indexes the two poles. It would be feasible to measure distance across the helmet surface in geodesic form, as discussed by Ventrucci *et al.* (2011), but for convenience simple Euclidean distance on the flattened brain map has been used here.

The relationship between the two parts of the dipole can be expressed in polar co-ordinates by defining the individual locations as:

$${}_{x,1}{}_{x}r\cos,\;\;\;{}_{y,1}{}_{y}r\sin{}_{x,2}{}_{x}r\cos,\;\;\;{}_{y,2}{}_{y}r\sin$$

where (*μ_x_*, *μ_y_*) denotes the centre of the dipole, θ denotes its orientation and *r* denotes the separation distance of the two poles. This assumes that the two poles have the same shape and the same size *h*. In order to avoid excessive overlap and thereby maintain a suitable dipole pattern, *r* should not exceed 2*h*.

### 3.2 Modelling the quasi-periodic temporal pattern

The general form of the dipole model in (3.1) modifies the fixed spatial patterns for the two poles, φ_1_ and φ_2_, by temporal weight functions γ_1_(*t*) and γ_2_(*t*). These are smooth functions of time which allow the size of each pole to vary and can therefore, in particular, characterize the oscillation of the dipole.

In practice, brain signals exhibit quasi-periodic rather than periodic oscillations, as illustrated in the real data displayed in Figure 1. Here, in order to display the underlying signal more clearly, the data have been smoothed in a similar manner to that described by Ventrucci *et al.* (2011), using 50 ‘effective degrees of freedom’ for space and 40 for time. The left hand plot shows a brain map with two sets of sensors highlighted, while the right hand plot shows the MEG signals at these highlighted locations. The shading on the left hand plot indicates the spatial pattern at a time snapshot. The plot of the highlighted sensors over time exhibits the typical oscillating behaviour of a dipole. As there are approximately five cycles within half a second, the dominant frequency is clearly in the α-band range of 8–12 Hz. However, there are several features worth noting.

- The signals at each pole do not have constant frequency. The behaviour is therefore *quasi-periodic* rather than perfectly periodic.
- The signals from the two poles are not out-of-phase for the whole timescale, as the phase shift changes with time. This is a common feature of dipoles which is due to their transient nature.
- The amplitude of the oscillations changes over time, both within and between the two poles.

These features require a strong degree of flexibility in the temporal weight functions γ_1_ and γ_2_. In addition to accommodating these features within the model, the aim is to quantify effects such as changes in amplitude and phase, so that the trial-to-trial variation in the behaviour of dipoles can be identified and characterized.

Eilers (2010) proposed a model for the smooth complex logarithm of an observed signal which is assumed to be the composition of two smooth functions over time. This approach can be adapted to the dipole setting by expressing the weight functions as:

(3.3) $${}_{i}(t)\exp({}_{i}(t\mathrm{))}\cos({}_{i}(t\mathrm{))}$$

where α(*t*) denotes amplitude on a log scale and ϕ(*t*) denotes phase. There are two steps in avoiding identifiability issues in model (3.3). The first is to express the amplitude curves as exp(*α_i_*(*t*)), so that they capture the size of the quasi-periodic signal in absolute terms. The second is to use the time points at which the temporal weight functions pass through 0 (‘zero-crossings’) to provide the crucial information on the phase *ϕ_i_*(*t*) of the quasi-periodic cycles, as discussed by Eilers (2010). The phase functions must also be non-decreasing. These issues will be revisited in the context of estimation, discussed in Section 4 below.

Finally, (3.3) is extended to the model:

(3.4) $${}_{i}(t){}_{i}(t)\exp\left({}_{i}(t)\right)\cos({}_{i}(t\mathrm{))}$$

by the introduction of a smooth intercept term *d_i_*(*t*). This allows possible shifts in the mean level of the signal, due to artifacts and noise, to be tracked. The issues involved in estimating the intercept function and the other terms of the model are discussed in Section 4 below.

## 4 Estimation

The process of model fitting is most easily approached in two stages. The first stage involves estimation of the two-dimensional spatial location, orientation and size of the dipole, as well as the temporal weight functions γ_1_ and γ_2_. The second stage uses ${\overset{}{}}_{1}$ and ${\overset{}{}}_{2}$ to construct estimates of the intercept, phase and amplitude of the dipole over time. These latter objects are the principal focus of the modelling process, as they characterize the dipole in a flexible but interpretable form and, in particular, allow changes over time to be tracked in a simple manner.

### 4.1 Estimation of the spatial parameters and temporal weights

The first stage involves estimation of the four spatial parameters defining the centre (*μ_x_*, *μ_y_*), orientation θ, radial size *h* and separation distance *r*, as well as the temporal weight functions γ_1_ and γ_2_, using the notation introduced in Section 3.1. Minimization of the sum-of-squares ${}_{j}\;{}_{k}\;(z_{\mathrm{jk}}m(x_{j},y_{j},t_{k}{\mathrm{))}}^{2}$, where *z_jk_* denotes the observed signal at sensor *j* and time point *k*, clearly involves non-linear optimization. However, the problem has a partially linear structure which can be exploited, since for fixed values of the spatial parameters, the model (3.1) is linear in γ_1_ and γ_2_. The fitting strategy described below therefore involves estimation of γ_1_ and γ_2_ nested within an optimization over the four spatial parameters, to achieve a global minimum of the sum-of-squares function.

If the MEG signals over space and time are structured as a vector ***z,*** then the model can be expressed in vector-matrix form using Kronecker products as

(4.1) $$z(I_{T}{}_{2}){}_{1}(I_{T}{}_{2}){}_{2}ɛ$$

where ***φ***_1_ and ***φ***_2_ are vectors of length *S*(= 248) which define the spatial pattern of the poles across the sensors, ***γ***_1_ and ***γ***_2_ are vectors of length *T*(= 128) which denote the value of the temporal weight functions for the two poles at the observed time points and ***ε***> is a vector of errors. This can be expressed in standard linear model form as zXɛ, with design matrix $X[I_{T}{}_{1},I_{T}{}_{2}]$ and vector of parameters $({}_{1}^{},{}_{2}^{}{)}^{}$.

A simple linear model could be fitted to the data from each separate time point. However, the spatiotemporal model (4.1) allows the estimation of γ to be improved by exploiting the assumption of smooth evolution of the underlying dipole over time. A simple strategy is to add to the sum-of-squares function $(zX{)}^{}(zX)$ a penalty term which will induce smoothness over time. This is a well established approach which is discussed in Eilers and Marx (1996) and many others papers. The smoothness of ***γ*** can be quantified by ${}^{}P$, where $PI_{2}D^{}D$ and the matrix *D* constructs second-order differences of the elements of ***γ***_1_ and ***γ***_2_. This leads to the penalized least squares solution:

(4.2) $$\overset{}{}(X^{}XP{)}^{1}X^{}z.$$

Note that this makes the reasonable assumption of a common penalty parameter λ for each half of the dipole. The formation of $X^{}X$ can be carried out very efficiently by exploiting the sparse nature of *X* while the solution of (4.2) can be efficiently computed through Cholesky decomposition.

The choice of smoothing parameter λ is an important issue. It is more convenient to address this on the more interpretable scale of the ‘effective degrees of freedom’ (edf) of the smoothing operation. This is easily calculated as the trace of the hat matrix $X(X^{}XP{)}^{1}X^{}$, or equivalently the trace of $(X^{}XP{)}^{1}X^{}X$, as discussed by Hastie and Tibshirani (1990), Wood (2006) and many others. Knowledge of the brain imaging context provides valuable information to inform a suitable choice of edf. For example, the oscillating frequency of dipoles occurring in the pre-stimulus period in the visual cortex is well known to be at the α-band of 8–12 Hz, as discussed by Van Dijk *et al.* (2008). For the range of data in the present application, a value of 60 edf was used in order to focus on frequencies of this order. This selection is justified by observing that for 10 Hz frequency we expect approximately five cycles over a period of 0.5 seconds, and so 60 edf allows modest flexibility of 6 edf for each cycle for each pole. This is further supported by empirical evaluation in Section 5. The value of λ which produces this specified value of edf can then be identified by a simple search.

The first stage of estimation can then be completed by minimizing the sum-of-squares from the penalized regression over the spatial parameters (*μ_x_*, *μ_y_*), θ, *r* and *h*. With non-linear optimization, starting values for the parameters can be very important. A very good preliminary estimate can be obtained by minimizing over discrete sets of parameter values which cover the full range of interest. Specifically, (*μ_x_*, *μ_y_*) ranged over a systematically placed subset of half of the sensor locations, θ over the values {0,/4,/2,3/4} and *h* over nine equally spaced values in the range 1 to 25 cm. This range of values for *h* covers all the realistic dipole sizes in relation to the physical area of the scalp. The separation distance parameter was set to 2*h* and *h*, with both of these values ensuring a characteristic dipole shape. The minimizing values of the parameters were then used as the starting point for a more general optimization algorithm to locate the final estimates on a continuous scale in the neighborhood of the initial values, using the Optim function in the R computing system (R Development Core Team, 2011).

### 4.2 Estimation of the quasi-periodic characteristics

Although the location and size of the dipole is of interest, there is particular value in characterizing dipole behaviour through the decomposition of the temporal weight function described in model (3.4), expressed in intercept *δ_i_*(*t*), amplitude *α_i_*(*t*) and phase *ϕ_i_*(*t*) curves over time. The quasi-periodic behaviour which dipoles exhibit requires flexible descriptions of each of these components and smoothing techniques such as p-splines, described by Eilers and Marx (1996) and many other authors, again provide suitable modelling tools. Specifically, a basis of regression functions is constructed from a set of overlapping B-spline functions, expressed in linear model form as ***δ_i_*** = B***d_i_, α_i_*** = B***a_i_*** and ***ϕ_i_*** = B***f_i_***, where ***δ_i_, α_i_*** and ***ϕ_i_*** represent the corresponding functions evaluated at the observed time points *t_k_*, *k* = 1, …, *T* and ***d****_i_*, ***a****_i_* and ***f****_i_* are vectors of B-spline coefficients. Each column of *B* has length *T* and is constructed by evaluating each B-spline basis function at the observed time points. Smoothness is induced through the use of second-order difference penalties, with specified edf.

The intercepts ***δ_i_***, which are required to track shifts in the signal, can be estimated by applying a p-spline smoothing procedure with low degrees of freedom to the estimates of ${\overset{}{}}_{i}$. Again, the expected form of the underlying signal offers guidance on suitable choices for the degree of smoothing. An α-band of 8–12 Hz will produce approximately 4 to 6 cycles during the pre-stimulus period of 0.5 seconds. An allocation of 1 edf for each cycle will ensure that the intercept term tracks the global movement in the mean signal rather than the individual cycles. In order to accommodate a small degree of further flexibility, a value of 7 edf was used, supported by some further sensitivity analysis.

The starting point for estimation of the phase and amplitude curves, *a_i_*(*t*) and *z_i_*(*t*), is the intercept-adjusted pole signal $p_{i}(t){}_{i}(t){}_{i}(t)$. As mentioned in Section 3.2, and following ideas discussed in Eilers (2010), the zero-crossings of *p_i_*(*t*) provide the crucial information on phase, because cos (ϕ(*t*)) is 0, and so the phase must be an odd multiple of *r*/2, at these locations. In practice, the zero-crossings ***γ****_i_* are identified by interpolation of ${\overset{}{}}_{i}{\overset{}{}}_{i}$, where the vector ***γ****_i_* has length 2*P* when there are *P* periods present. The phase curve *z_i_*(*t*) is then estimated by p-spline smoothing of the vector {/2,3/2,,(4P1)/2} against ***γ****_i_*. Information on phase can easily by translated onto the frequency scale by computing the empirical derivative of the estimated phase curve, scaled by 2*r*, namely:

(4.3) $${\overset{}{f}}_{i}(t)\frac{{\overset{}{{}^{}}}_{i}(t)}{2}.$$

Here, the derivatives were estimated very effectively by simple differencing although the piecewise polynomial algebra of B-splines provide a relatively straightforward alternative, as discussed by Boor (1978).

In a similar manner, again following Eilers (2010), a parsimonious expression of amplitude information is available through the logarithm of the intercept-adjusted pole signal *p_i_*(*t*) at the time points which lie mid-way between the zero-crossings. If these vectors are denoted by ***l_i_*** and ***m_i_*** respectively, then the amplitude curve *a_i_*(*t*) is estimated by p-spline smoothing of the vector ***l_i_*** against ***m_i_***.

Since the estimates ${\overset{}{}}_{i}(t)$ from which the intercept-phase-amplitude decomposition is being derived are already smooth quantities, expression of the large-scale structure in this point-based manner loses relatively little information. It does provide a compact representation of the characteristics of a complex temporal structure through estimated intercept, phase and amplitude functions.

For the degree of smoothing, 3 edf were used for the point-based representations of both phase and amplitude. For phase, an exactly periodic pattern corresponds to linear phase and so an edf of 2. With the α-band signal over 0.5 seconds, we expect around 10 zero-crossings and so we allow a small degree of flexibility beyond linear. The amplitude was also estimated with 3 edf. In both cases we are interested in identifying large-scale changes, not in tracking small scale movement. A sensitivity analysis offers reassurance on the suitability of these choices.

Note that it would in principle be feasible to refine the estimates of phase and amplitude by Gauss-Newton iteration within the non-linear model (3.4), following Eilers (2010). However, in the present setting, where estimation is based on smooth quantities ${\overset{}{}}_{1}(t)$, ${\overset{}{}}_{2}(t)$ rather than noisy data, we found the initial estimates to be perfectly adequate in characterizing the underlying quasi-periodic behaviour.

## 5 Simulation study

We have precise information about the nature of the dipole that we want to extract from noisy single trial pre-stimulus data of the experiment under study. The brain activity associated with visual attention (the brain awaiting for a visual stimulus) is a dipole, with a certain unknown location, oscillating over time at a frequency expected to be in the 8–12 Hz range, referred to as the α-band frequency. In the first stage of our model fitting procedure we need to choose a value for the edf which is appropriate to fit this sort of dipole. In Section 4 we argued the choice of 60 degrees of freedom seems reasonable for modelling smooth pole signals of an α-band dipole operating over an interval of half a second, corresponding to the pre-stimulus period. In order to investigate the effectiveness of this choice we conducted a simulation study and evaluated the mean squared error of estimation for both amplitude and frequency curves of an α-band dipole. Four scenarios were considered.

- *Scenario 1.* A single α-band dipole is present, with topography as in panel α of Figure 1. The pole signals have constant amplitude and frequency over time.
- *Scenario 2:* A single α-band dipole is present, with topography as in panel α Figure 1, but the pole signals have amplitudes and frequencies which increase over time.
- *Scenario 3:* One dominant α-band dipole is present as described in Scenario 2, but another α-band dipole of lower amplitude is also present, with topography as in panel γ of Figure 1. The minor dipole has constant amplitude and frequency.
- *Scenario 4:* One dominant α-dipole and one minor α-dipole are present, as described in Scenario 3, but in addition there is a trend in the mean over time, reflecting an artifact.

**Figure 2:**
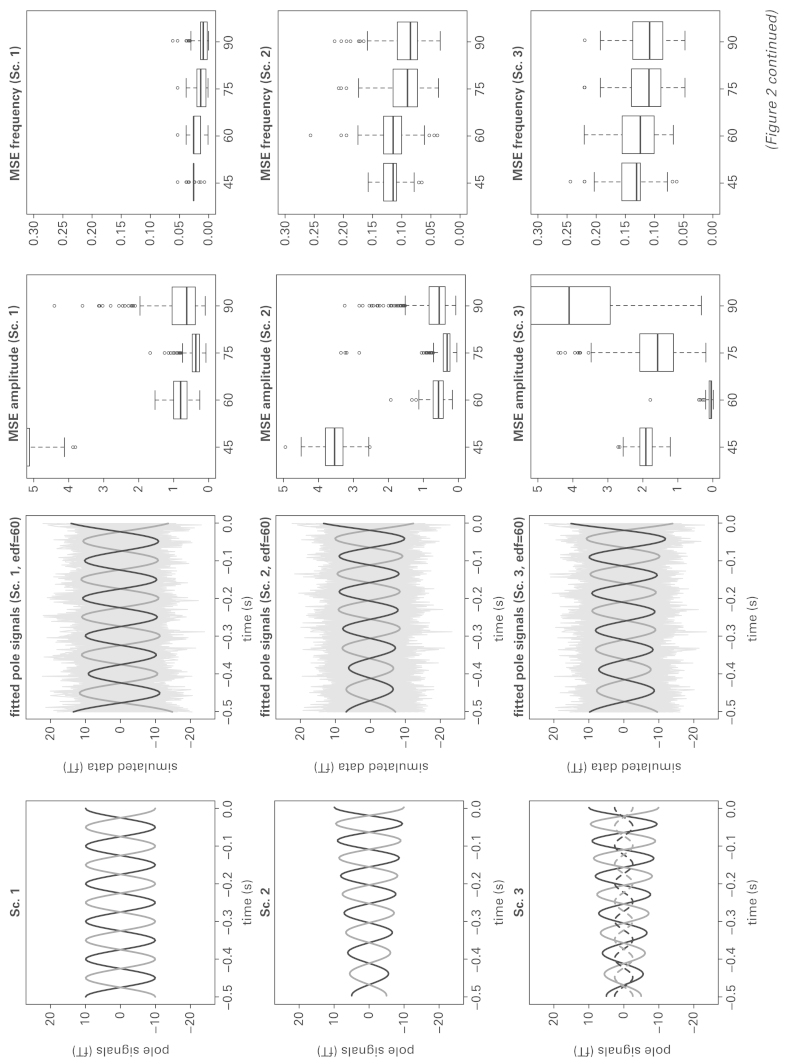

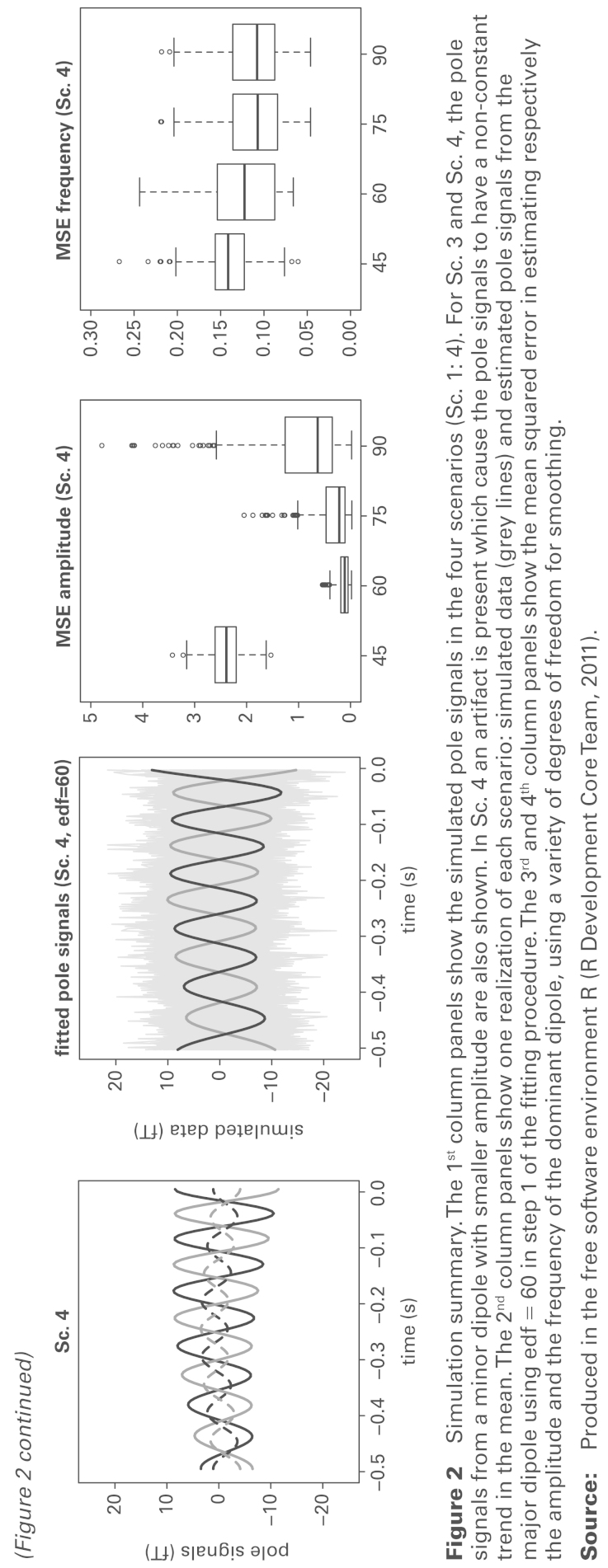
Simulation summary. The 1^st^ column panels show the simulated pole signals in the four scenarios (Sc. 1: 4). For Sc. 3 and Sc. 4, the pole signals from a minor dipole with smaller amplitude are also shown. In Sc. 4 an artifact is present which cause the pole signals to have a non-constant trend in the mean. The 2^nd^ column panels show one realization of each scenario: simulated data (grey lines) and estimated pole signals from the major dipole using edf = 60 in step 1 of the fitting procedure. The 3^rd^ and 4^th^ column panels show the mean squared error in estimating respectively the amplitude and the frequency of the dominant dipole, using a variety of degrees of freedom for smoothing. **Source:** Produced in the free software environment R (R Development Core Team, 2011).

These four scenarios present increasing challenges in estimating the phase and amplitude components of the dominant dipole. Scenarios 3 and 4 are the most challenging because of the presence of a minor dipole which may corrupt estimation of the temporal and spatial pattern of the dominant one. Even worse, the minor dipole oscillates in the same α-band frequency as the dominant one. However, this is a situation that can frequently be met in practice. In Figure 2, the panels in the first column display the true pole signals in each of the four scenarios as grey and black lines. The panels in the second columns show the noisy MEG data as grey lines, simulated by constructing the Kronecker product of the true dipole topography with the true pole signals and adding random noise at each sensor and time point to reflect model (4.1).

Model (3.1) was fitted to each of 500 realizations from each scenario, using several choices of effective degrees of freedom, edf = {30, 45, 60, 75, 90}. Our modelling aim is to identify the dominant dipole source in the data, unaffected by any minor source. In Figure 2, the panels in the third and fourth columns show boxplots of the mean squared errors (*MSE*) in estimation of the amplitude and frequency curves of the dominant dipole, using a variety of edf values. The level of smoothing, expressed in edf, has a strong impact on amplitude estimation, while frequency estimation is relatively unaffected. For amplitude, in Scenario 3 where a minor dipole is present the use of 60 edf produces a performance which is markedly superior to other degrees of smoothing. This remains the best choice in Scenario 4, while it is close to optimal in Scenarios 2 and 3. For frequency, the results are much less affected by the edf value. These results therefore provide strong empirical evidence of the suitability of 60 edf for identifying the size and nature of the evolution of an α-band dipole. Smaller values of edf are less effective in tracking the temporal pattern while larger values are subject to greater variability and may be more strongly influenced by subsidiary dipoles. The use of 60 edf is seen to provide an effective and stable choice.

**Figure 3:**
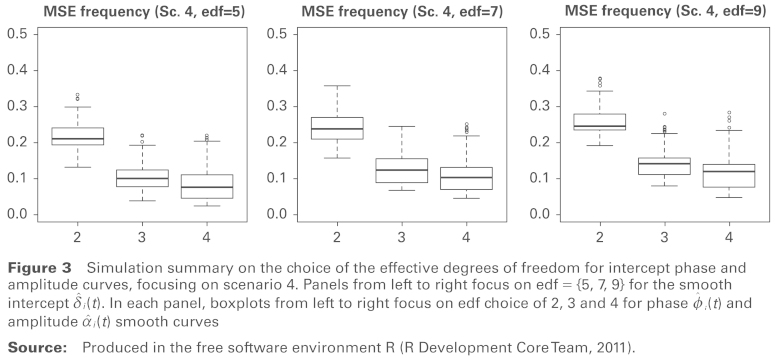
Simulation summary on the choice of the effective degrees of freedom for intercept phase and amplitude curves, focusing on scenario 4. Panels from left to right focus on edf = {5, 7, 9} for the smooth intercept ${\overset{}{}}_{i}(t)$. In each panel, boxplots from left to right focus on edf choice of 2, 3 and 4 for phase ${\overset{}{}}_{i}(t)$ and amplitude ${\overset{}{}}_{i}(t)$ smooth curves **Source:** Produced in the free software environment R (R Development Core Team, 2011).

Simulation was also used to check the robustness of the choice of edf for the estimation of the intercept, amplitude and phase curves in the second stage of the fitting procedure. In particular, the choice of the degree of smoothness for the intercept curve ${\overset{}{}}_{i}(t)$ is important in effective identification of zero-crossings and consequent estimation of the phase and frequency curves. The three panels of Figure 3 display MSE for estimation of the frequency curve for Scenario 4, using edf = {5, 7, 9} in estimation of the intercept curve. Within each panel the boxplots report the MSE in estimation of the frequency curve using edf = {2, 3, 4} for both phase and amplitude curves. The principal beneficial effect is in allowing the phase and amplitude curve to be more flexible than a simple linear pattern by ensuring that the edf is larger than 2. Beyond that, there is relatively little change in the use of higher values. This again provides empirical evidence to support the use of 7, 3 and 3 edf for the intercept, phase and amplitude curves.

## 6 Application

The dipole model and its associated fitting procedure were applied to the pre-stimulus data from the MEG experiment described in Section 2. In order to assess the effectiveness of the approach, a model for a single trial on a single subject is first examined in detail. The model is then applied to all available trials for several individuals to gain insight into the nature of trial-to-trial variability, both within and across individuals.

### 6.1 Analysis of a single-trial

**Figure 4:**
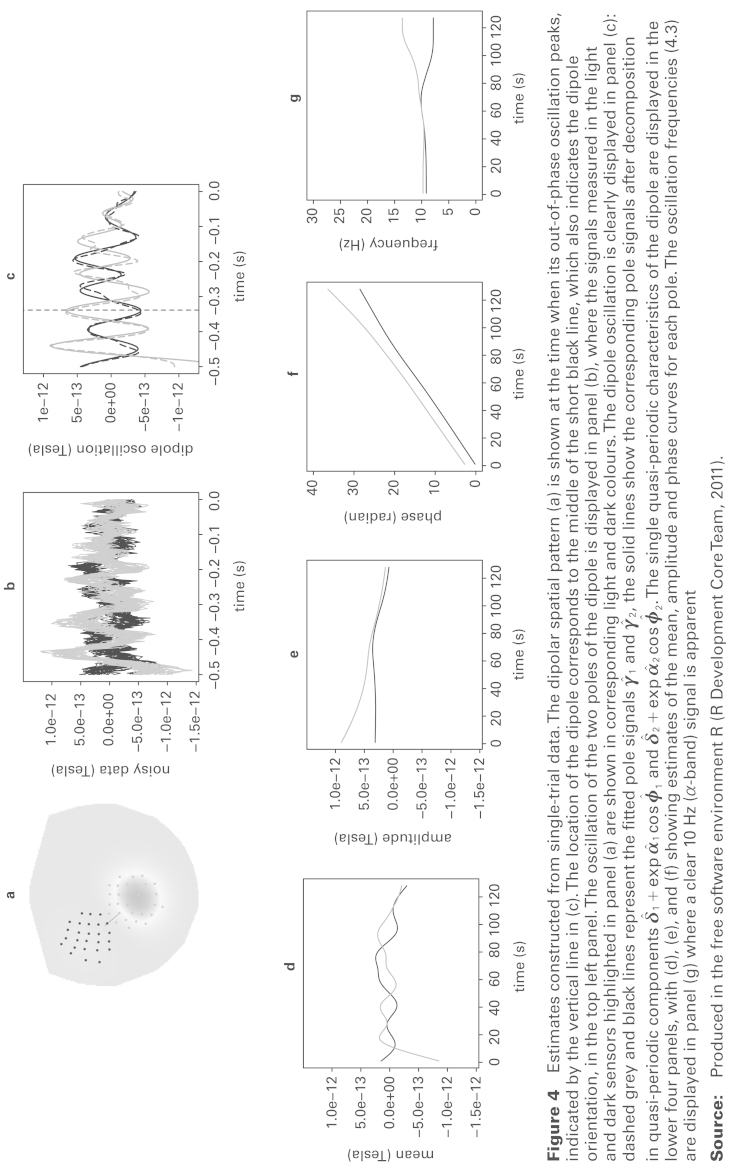
Estimates constructed from single-trial data. The dipolar spatial pattern (a) is shown at the time when its out-of-phase oscillation peaks, indicated by the vertical line in (c). The location of the dipole corresponds to the middle of the short black line, which also indicates the dipole orientation, in the top left panel. The oscillation of the two poles of the dipole is displayed in panel (b), where the signals measured in the light and dark sensors highlighted in panel (a) are shown in corresponding light and dark colours. The dipole oscillation is clearly displayed in panel (c): dashed grey and black lines represent the fitted pole signals ${\overset{}{}}_{1}$ and ${\overset{}{}}_{2}$, the solid lines show the corresponding pole signals after decomposition in quasi-periodic components ${\overset{}{}}_{1}\exp{\overset{}{}}_{1}\cos{\overset{}{}}_{1}$ and ${\overset{}{}}_{2}\exp{\overset{}{}}_{2}\cos{\overset{}{}}_{2}$. The single quasi-periodic characteristics of the dipole are displayed in the lower four panels, with (d), (e), and (f) showing estimates of the mean, amplitude and phase curves for each pole. The oscillation frequencies (4.3) are displayed in panel (g) where a clear 10 Hz (α-band) signal is apparent **Source:** Produced in the free software environment R (R Development Core Team, 2011).

Results from a single trial are displayed in Figure 4. The spatial information in the top left hand panel has identified a dipole on the left side of the motor cortex. The estimates of γ_1_(*t*) and γ_2_(*t*) in the top right hand panel show the two parts of the dipole to be oscillating strongly out-of-phase, over the initial time period at least. The raw MEG signals from sensors inside the poles (marked in the top left hand plot) are displayed in the top middle panel to confirm that the smoothing procedure has removed high frequency noise and summarized the temporal patterns effectively.

The four lower panels show the functions derived from γ_1_(*t*), γ_2_(*t*) which characterize the temporal nature of the dipole. The estimates of the intercept and (log) amplitude curves which are displayed in the first two panels show effectively constant means for each pole and only minor differences in amplitude in the early part of the pre-stimulus period. The second two panels show the information on phase, together with the instantaneous frequency curves. These confirm that we are indeed examining information from the α-band 8–12 Hz range with the two halves of the dipole oscillating at the same rate until a small degree of divergence appears immediately before the stimulus is applied.

The physics of current flow determines that a genuine dipole is present when the two separate poles are completely out-of-phase and so the difference in phase provides the key information to assess this. More precisely, the quantity:

(6.1) $$(t)\min((t\mathrm{),}2(t\mathrm{)),}\;\mathrm{where}\;(t)|{\overset{}{}}_{1}(t){\overset{}{}}_{2}(t\mathrm{)|}\mathrm{mod}2,$$

gives a curve which takes values over the interval [0, π] and which takes the value π when the two poles are exactly out-of-phase. With this particular trial, β(*t*) (not shown) lies close to π for the first 300ms and diminishes thereafter, giving a clear indication of the presence of a transient dipole, indicating the alert state of the brain while a visual stimulus is awaited. The peak of β(*t*) is identified by the vertical line in the top right panel of Figure 4.

Further work would allow standard errors to be computed for the curves which characterize the behaviour of a dipole, along the lines of the analysis described by Ventrucci *et al.* (2011). However, the focus of the present article is the variability across trials and so the estimates which have been derived are used to examine and describe the random variation at trial level.

### 6.2 Functional data analysis of many trials

When viewing the results of fitted models for a large number of trials, a more systematic and quantitative method of identifying the presence of a dipole is required. Two conditions must be satisfied, namely that the poles oscillate at the same frequency and that they are fully out-of-phase. A simple empirical rule might identify a dipole if $|{\overset{}{f}}_{1}(t){\overset{}{f}}_{2}(t\mathrm{)|}<2$ and (t)<1. Of the 384 trials examined for instance for subject 1, this rule identifies a dipole within the α-band range at some time point in 307 cases. However, it is more appealing to quantify the strength of evidence for a dipole in a more continuous manner and this can be achieved by use of the weight function:

$$w(t)\exp\{0.5({\overset{}{f}}_{i}(t){\overset{}{f}}_{2}(t{\mathrm{))}}^{2}/1^{2}\}\;\exp\{0.5((t{\mathrm{))}}^{2}/0.5^{2}\mathrm{\},}$$

which decreases from 1 in a smooth manner as ${\overset{}{f}}_{1}(t)$ and ${\overset{}{f}}_{2}(t)$ move apart and as β(*t*) moves away from π. The ‘standard deviation’ parameters of the exponential components have been chosen to match the simple binary rule given above.

**Figure 5:**
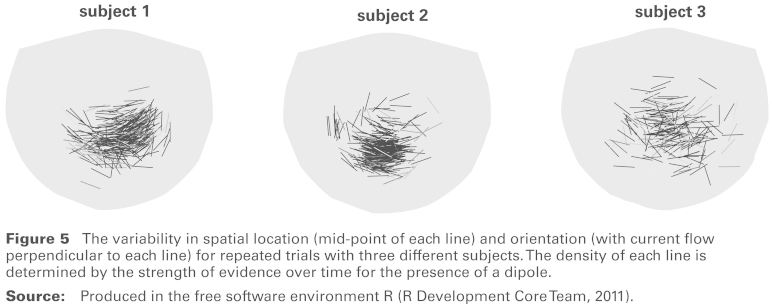
The variability in spatial location (mid-point of each line) and orientation (with current flow perpendicular to each line) for repeated trials with three different subjects. The density of each line is determined by the strength of evidence over time for the presence of a dipole. **Source:** Produced in the free software environment R (R Development Core Team, 2011).

Figure 5 shows the spatial distribution of the dipoles detected in all the available trials for each of three subjects involved in the experiment. The lines connect the two poles of the dipole and their mid-points represent the estimated dipole locations. The density of each line is determined by the maximum of the weight function *w*(*t*) over time, so that lightly shaded lines indicate weaker evidence for the presence of a dipole. There is a striking dependence between the spatial location and orientation of the dipole in subjects 1 and 2. This is indicative of brain anatomy, as variation in the dipole location is constrained by the topography of the folds in brain tissue. The size of the spatial variation is similar across the subjects, although subject 3 shows a slightly more diffused pattern.

**Figure 6:**
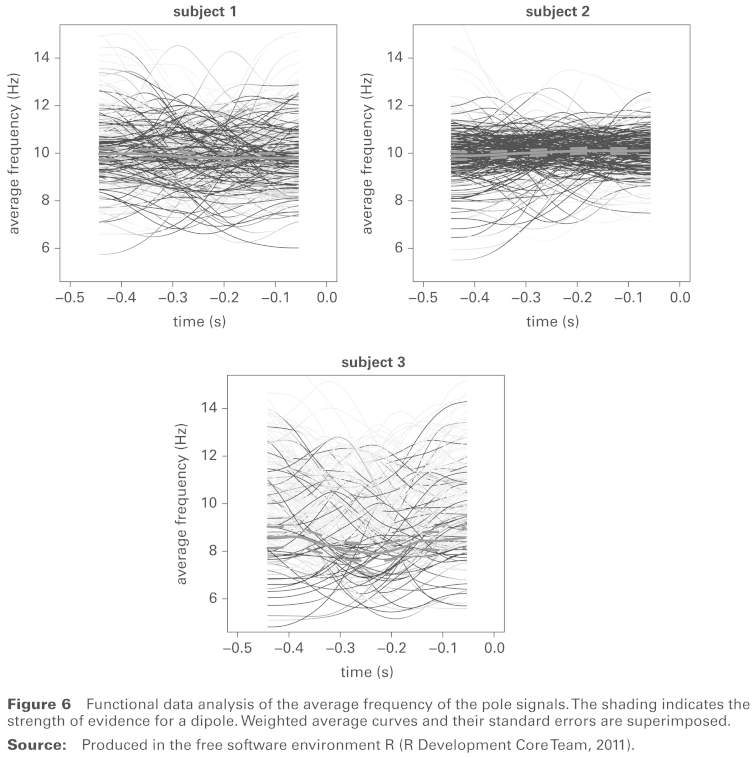
Functional data analysis of the average frequency of the pole signals. The shading indicates the strength of evidence for a dipole. Weighted average curves and their standard errors are superimposed. **Source:** Produced in the free software environment R (R Development Core Team, 2011).

Figure 6 plots the frequency information for each trial, averaged over the two parts of the dipole, denoted by ${\overset{}{f}}_{j}(t)$. Again, shading has been used to indicate the strength of evidence for a dipole, with the grey-scale of curve *j* at each time *t* determined by the corresponding value of *w_j_*(*t*). The large number of individual trials makes underlying patterns difficult to assess, so a mean curve across trials was computed by taking a weighted average of the ${\overset{}{f}}_{j}(t)$, using the *w_j_*(*t*) as weights. The mean curves are at the expected 10 Hz for subjects 1 and 2 but at the lower end of the α–band for subject 3. In addition, there are clear differences in the size of the variation around these mean values, with subject 2 displaying high concentration of the individual trials, while subject 3 in particular has much greater variation. These characteristics of different subjects are missed by methods of analysis based on averaging over trials.

**Figure 7:**
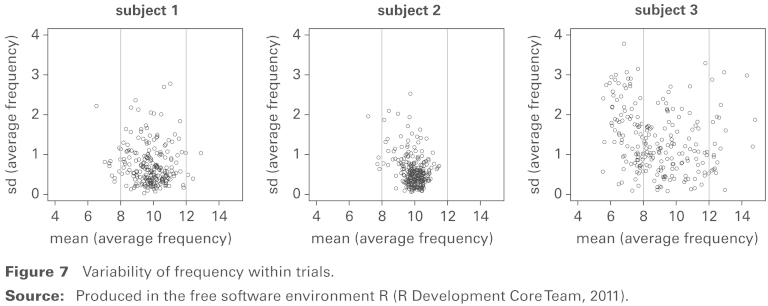
Variability of frequency within trials. **Source:** Produced in the free software environment R (R Development Core Team, 2011).

There is particular interest in the size of the variability in the frequency within each trial, and Figure 7 explores this by plotting the standard deviation over time of ${\overset{}{f}}_{j}(t)$ against the mean over time of ${\overset{}{f}}_{j}(t)$. Again, subjects 1 and 2 show high degrees of concentration at the characteristic α–band frequency of 10 Hz while subject 3 exhibits greater variability. Within this subject, variability is smaller within the α–band frequency range 8–12 Hz than outside it.

Overall, these results demonstrate significant variability of frequency of brain oscillations within and between participants and they question the validity of the conventional averaging approach across trials and participants. Instead, it has been demonstrated that model-based analysis allows single-trial estimation of time-varying amplitude, phase and frequency and makes it possible to relate the trial-by-trial variation of these measures to trial-by-trial variations in behavioural performance, such as perceptual accuracy or reaction time.

## 7 Discussion

Traditionally, oscillations in MEG signals are analyzed by means of temporal band-pass filters or Fourier transforms. Both techniques rely on an assumption of stationarity of the underlying signal. However, brain activity is highly dynamic and non-stationary, with brain oscillations typically showing variation in both amplitude and frequency over time, and across trials. The methods proposed in this article aim to adapt to these features and, importantly, to allow the robust estimation of amplitude, frequency and phase dynamics on single trial data. Recent research has demonstrated that amplitude, phase and frequency contain important information about the state of the human brain that correlates with behavioural performance. Investigation of this requires models and methods that are adapted to the complex dynamic nature of single trial signals.

The nature of the variation across trials and across individuals provides valuable insight which is not available from analysis based on averaged data. In addition to the direct interpretation about the nature of brain signals, this random variation also provides the building blocks from which random effect models involving the comparison of treatment groups, and more complex designs, may be constructed.

The simulation study showed that estimation of a dominant dipole is not adversely affected by the presence of other minor dipoles. However, there is interest in simultaneously identifying multiple dipoles, especially in the post-stimulus period when there may be multiple simultaneously activated brain areas of interest. In future work, this will be addressed by building in more explicit representations of the mapping from a dipole source at a three-dimensional position within the human brain to the corresponding spatial field pattern on the sensor surface. This mapping can be constructed by considering the underlying physics and it could be the basis of disambiguating contributions from different activated brain areas.
